# Association Between Proton Pump Inhibitor Use and Short-Term Postoperative Complications Following Abdominoplasty: A Multi-Institutional Cohort Study

**DOI:** 10.1093/asjof/ojag129

**Published:** 2026-06-23

**Authors:** Yoram Wolf, Jonathan Zontag, Ron Skorochod, Nir Zontag

## Abstract

**Background:**

Abdominoplasty is a common procedure, especially among patients with obesity, a group in which proton pump inhibitor (PPI) use is highly prevalent. Although there are growing concerns regarding PPI-related complications, there is a lack of data on their effects in aesthetic surgery.

**Objectives:**

This study aims to assess the association between preoperative use of PPIs and the risk of short-term postoperative complications after abdominoplasty.

**Methods:**

This multicenter cohort study used the TriNetX Global Collaborative Network. Individuals aged 18 years or older who had abdominoplasty were identified and categorized into 2 groups based on preoperative PPI exposure: patients with a documented prescription before surgery and those without any recorded history of PPI prescription. To reduce baseline differences between groups, propensity score matching was conducted to achieve balance in demographic and clinical characteristics. Postoperative outcomes focused on complication rates evaluated at 30, 60, and 90 days after surgery.

**Results:**

After 1:1 propensity score matching, both groups comprised 2031 patients each. At 30 days following abdominoplasty, patients who received PPIs before surgery exhibited significantly higher risks of surgical-site infections (risk ratio [RR] 2.467; 95% CI, 1.358-4.48), wound dehiscence (RR 2; 95% CI, 1.079-3.706), opioid prescription (RR 1.368; 95% CI, 1.269-1.475), and any surgical-site complications (RR 2.545; 95% CI, 1.806-3.588) compared with the control group. These risks persisted at 60 and 90 days postsurgery.

**Conclusions:**

Preoperative exposure to PPIs may increase the risk of short-term postoperative complications following abdominoplasty. Additional studies are needed to define clinical guidelines regarding the perioperative management of these medications.

**Level of Evidence:3 (Therapeutic):**

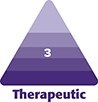

Proton pump inhibitors (PPIs) are among the most widely used acid-suppressive agents.^1^ These medications are commonly prescribed for conditions, such as gastroesophageal reflux disease and peptic ulcers. Furthermore, they are often used for long-term maintenance to prevent symptom recurrence.^1-3^ Although PPIs are generally considered “safe,” their use has been associated with various adverse effects, including fractures, nutrient deficiencies, dysbiosis, and subsequent infections.^4^

Importantly, many of these acid-related conditions are highly prevalent among individuals with obesity.^5^ Reports indicate that obesity doubles the risk of gastroesophageal reflux disease, affecting up to 50% of this patient's population.^5,6^

Recently, studies have identified several adverse effects associated with PPI use.^7-9^ Specifically, higher rates of complications such as postoperative infections, deep vein thrombosis, gastrointestinal bleeding, and mortality have been observed among patients prescribed PPIs before elective surgeries.^7,8^ Similarly, Bruin et al reported that PPI users had an increased risk of developing surgical-site infections following orthopedic joint surgeries.^9^

Abdominoplasty is a common aesthetic procedure, especially among individuals with current or past obesity. Because conditions treated with PPIs are highly prevalent in this population and given recent concerns regarding their potential effects on postoperative outcomes, we found it essential to evaluate whether patients who use PPIs are at increased risk for postoperative complications. In this study, we aim to assess the impact of PPI use on short-term postoperative complications following abdominoplasty, utilizing the TriNetX global electronic medical records database.

## METHODS

This study is a retrospective cohort study utilizing the TriNetX Global Collaborative Network (TriNetX LLC, Cambridge, MA). This network contains deidentified electronic health/medical records from 172 healthcare organizations (updated to December 2025).

### Ethics

All data accessed through the TriNetX network are anonymized in compliance with the Health Insurance Portability and Accountability Act (HIPAA) privacy standards. Because this analysis utilizes only previously collected, nonidentifiable information, Institutional Review Board and patient consent are not required under HIPAA. This analysis represents a secondary review of existing data, involves no intervention or interaction with human participants, and complies with the de-identification standard specified in Section §164.514(a) of the HIPAA Privacy Rule. The de-identification process is verified through a formal determination by a qualified expert, as defined in Section §164.514(b)(1) of the HIPAA Privacy Rule.

### Cohort Definition

Two cohorts of patients aged 18 years and older were defined. The first cohort consisted of patients who underwent abdominoplasty and documented prescriptions for PPIs within 1 year before surgery. The second cohort comprised patients who underwent the same procedure, had no history of PPI prescriptions, but received histamine 2 receptor antagonists (H2 blockers) during the same preoperative period. The index event was defined as the date of the procedure. Supplemental Table S1 provides the complete list of codes for exclusion and inclusion criteria.

Patients who were prescribed H2 blockers were selected as the control group because both PPIs and H2 blockers share similar primary clinical indications, such as gastroesophageal reflux disease and peptic ulcer disease.^10^ Using an active comparator with overlapping indications helps ensure that both cohorts have comparable underlying gastrointestinal conditions and healthcare utilization patterns, thereby reducing confounding related to indication and improving baseline comparability within the observational database.

### Outcomes Measured

The primary objective of this study was to assess short-term complications following abdominoplasty. Three-time intervals were defined: 30, 60, and 90 days postsurgery. We evaluated wound dehiscence, surgical-site infection, seroma, hematoma, opioid prescription, hematoma/seroma evacuation, inpatient hospitalization, readmission, and any surgical-site complications. TriNetX does not report outcomes with fewer than 10 patients, to protect patient privacy.

### Statistical Analysis

Propensity score matching at a 1:1 ratio was conducted using logistic regression, incorporating baseline characteristics from the year before the index date, such as medical conditions, demographics, and substance use. Variables were considered as significantly different if *P*-value was <.05. Supplemental Table S2 summarizes a list of all variables used to balance cohorts.

Statistical analyses were conducted solely using the TriNetX platform. For each outcome, risk ratios (RRs), and 95% CIs were calculated. Statistical significance was defined as a *P*-value of <.05. Continuous variables are presented as mean ± standard deviation (SD). Also, sex was documented where available. Sex-specific counts include only patients with documented sex, whereas patients with missing or unknown sex were included in the total cohort but excluded from sex-stratified subgroup analyses.

## RESULTS

Initially, 10,307 (mean age ± SD: 43.11 ± 10.89 years) patients were included in the PPI group, and 2824 patients (mean age ± SD: 44.18 ± 10.73 years) in the H2 group. After 1:1 propensity score matching, the 2 groups were balanced to 2031 each. The PPI group included 1906 females (93.8%) and 123 males (6.1%), whereas the H2 group included 1906 females (93.8%) and 124 males (6.1%). Demographics, comorbidities, and medication use did not differ significantly between groups (Table 1), except for a higher rate of White patients in the PPI group (72% in PPI group vs 65.9% in H2 group).

**Table 1.** Baseline Characteristics of Patients Undergoing Abdominoplasty, Comparing PPI Users With Control H2 Users

	Before matching		*P*-value	After matching		*P*-value
Characteristics, *n* (%)	PPI cohort (*n* = 10,307)	Control cohort (*n* = 2824)		PPI cohort (*n* = 2031)	Control cohort (*n* = 2031)	
Age at index, years (mean ± SD)	43.11 ± 10.89	44.18 ± 10.73	<.001*	44.21 ± 11.28	44.05 ± 10.92	.636
Female	9552 (92.7%)	2685 (95.1%)	<.001*	1906 (93.8%)	1906 (93.8%)	1.000
Male	751 (7.3%)	137 (4.9%)	<.001*	123 (6.1%)	124 (6.1%)	.948
White	3841 (37.3%)	1983 (70.2%)	<.001*	1463 (72.0%)	1338 (65.9%)	<.001*
Black or African American	486 (4.7%)	274 (9.7%)	<.001*	133 (6.5%)	153 (7.5%)	.220
Not Hispanic or Latino	1644 (16.0%)	1386 (49.1%)	<.001*	752 (37.0%)	740 (36.4%)	.696
Hispanic or Latino	275 (2.7%)	277 (9.8%)	<.001*	112 (5.5%)	130 (6.4%)	.233
Overweight and obesity	1322 (12.8%)	195 (6.9%)	<.001*	229 (11.3%)	191 (9.4%)	.050
Hypertensive diseases	969 (9.4%)	171 (6.1%)	<.001*	182 (9.0%)	161 (7.9%)	.236
Type 2 diabetes mellitus	451 (4.4%)	89 (3.2%)	.004*	87 (4.3%)	83 (4.1%)	.754
Type 1 diabetes mellitus	24 (0.2%)	10 (0.4%)	.261	10 (0.5%)	10 (0.5%)	1.000
Chronic kidney disease	87 (0.8%)	13 (0.5%)	.038*	12 (0.6%)	10 (0.5%)	.669
Gastroesophageal reflux disease	1164 (11.3%)	78 (2.8%)	<.001*	94 (4.6%)	78 (3.8%)	.213
Peptic ulcer disease	20 (0.2%)	10 (0.4%)	.114	10 (0.5%)	10 (0.5%)	1.000
Hematemesis	10 (0.1%)	10 (0.4%)	.002*	10 (0.5%)	10 (0.5%)	1.000
Chronic obstructive pulmonary disease	75 (0.7%)	10 (0.4%)	.028*	10 (0.5%)	10 (0.5%)	1.000
Nicotine dependence	96 (0.9%)	21 (0.7%)	.347	29 (1.4%)	18 (0.9%)	.107
BMI, kg/m^2^ (mean ± SD)	30.24 ± 5.57	28.81 ± 5.72	<.001*	29.73 ± 5.34	29.40 ± 5.64	.216
BMI 30-35 kg/m^2^	944 (9.2%)	475 (16.8%)	<.001*	336 (16.5%)	334 (16.4%)	.933
BMI 35-40 kg/m^2^	483 (4.7%)	199 (7.0%)	<.001*	147 (7.2%)	144 (7.1%)	.855
BMI 40-45 kg/m^2^	191 (1.9%)	69 (2.4%)	.046*	52 (2.6%)	48 (2.4%)	.685
BMI ≥45 kg/m^2^	95 (0.9%)	28 (1.0%)	.733	27 (1.3%)	18 (0.9%)	.177

Baseline characteristics of patients undergoing abdominoplasty are compared between those receiving preoperative PPIs and the control group receiving H2 blockers. Continuous variables are presented as means ± SD, whereas categorical variables are shown as counts (percentages).

PPI, proton pump inhibitor; SD, standard deviation.

^*^Statistically significant.

At 30 postoperative days, patients who received preoperative PPIs demonstrated significantly increased rates of surgical-site complications, corresponded to RR 2.545 (95% CI, 1.806-3.588; *P* < .001), including wound dehiscence (RR 2; 95% CI, 1.079-3.706; *P* = .028) and surgical-site infection (RR 2.467; 95% CI, 1.358-4.48; *P* = .003) comparing to the H2 control patients. Additionally, patients in the PPI group had higher rates of opioid prescription (RR 1.368; 95% CI, 1.269-1.475; *P* < .001). However, no significant risk for inpatients hospitalization (*P* = .334), readmission (*P* = .16), or hematoma/seroma evacuation (*P* = .06) were noted. Table 2 summarizes the complete outcomes measured.

**Table 2:** Thirty-Day Postoperative Outcomes in Patients PPI Users vs Control H2 Users Patients

	PPI cohort (*n* = 2031)	Control cohort (*n* = 2031)	RR	95% CI	*P*-value
Surgical-site infection	37	15	2.467	1.358-4.48	.003*
Wound dehiscence	30	15	2	1.079-3.706	.028*
Hematoma	No. of events was too small	No. of events was too small	NA	NA	NA
Seroma	No. of events was too small	No. of events was too small	NA	NA	NA
Readmission	20	12	1.667	0.817-3.4	.160
Inpatient hospitalization	60	50	1.2	0.829-1.738	.334
Hematoma/seroma evacuation	22	11	2	0.972-4.114	.060
Opioid prescription	963	704	1.368	1.269-1.475	<.001*
Any surgical-site complications	112	44	2.545	1.806-3.588	<.001*

Thirty-day postoperative outcomes for patients undergoing abdominoplasty are compared between those receiving preoperative proton pump inhibitors (PPIs) and a control group receiving H2 blockers. Outcomes are reported as risk ratios (RRs) with 95% CIs.

^*^Statistically significant.

After 60 days of surgery, PPI group showed an increased risk for surgical-site complications (RR 2.227; 95% CI, 1.677-2.958; *P* < .001), including surgical-site infection (RR 2.30; 95% CI, 1.366-3.874; *P* = .002), seroma (RR 2.214; 95% CI, 1.182-4.150; *P* = .013), and hematoma/seroma evacuation (RR 1.895; 95% CI, 1.091-3.292; *P* = .023) in comparison with control H2 group. Similar to the observation at 30 days, opioid prescription remained increased in the PPI cohort (RR 1.388; 95% CI, 1.289-1.495; *P* < .001; Table 3).

**Table 3:** Sixty-day Postoperative Outcomes in Patients PPI Users vs Control H2 Users Patients

	PPI cohort (*n* = 2031)	Control cohort (*n* = 2031)	RR	95% CI	*P*-value
Surgical-site infection	46	20	2.300	1.366-3.874	.002*
Wound dehiscence	43	28	1.536	0.958-2.462	.075
Hematoma	10	11	0.909	0.387-2.136	.827
Seroma	31	14	2.214	1.182-4.150	.013*
Readmission	23	13	1.769	0.899-3.483	.099
Inpatient hospitalization	61	52	1.173	0.815-1.689	.391
Hematoma/seroma evacuation	36	19	1.895	1.091-3.292	.023*
Opioid prescription	987	711	1.388	1.289-1.495	<.001^*^
Any surgical-site complications	147	66	2.227	1.677-2.958	<.001*

Sixty-day postoperative outcomes for patients undergoing abdominoplasty are compared between those receiving preoperative proton pump inhibitors (PPIs) and a control group receiving H2 blockers. Outcomes are reported as risk ratios (RRs) with 95% CIs.

^*^Statistically significant.

Following 90 days, abdominoplasty patients who were prescribed PPIs continued to demonstrate significantly higher risk of postoperative complications. Surgical-site complications were increased (RR 2.31; 95% CI, 1.761-3.031; *P* < .001), specifically surgical-site infection (RR 2.364; 95% CI, 1.441-3.876; *P* = .001) and wound dehiscence rates (RR 1.633; 95% CI, 1.041-2.562; *P* = .033), when compared with matched H2 control patients.

Moreover, significantly higher rates of seroma (RR 2.0; 95% CI, 1.101-3.633; *P* = .023) and subsequent hematoma/seroma evacuation (RR 2.0; 95% CI, 1.157-3.457; *P* = .013) were observed. Higher rates of readmission (RR 2.077; 95% CI, 1.075-4.014; *P* = .03) and opioid prescription (RR 1.390; 95% CI, 1.292-1.496; *P* < .001) were also observed (Table 4).

**Table 4:** Ninety-day Postoperative Outcomes in Patients PPI Users vs Control H2 Users Patients

	PPI cohort (*n* = 2031)	Control cohort (*n* = 2031)	RR	95% CI	*P*-value
Surgical-site infection	52	22	2.364	1.441-3.876	.001*
Wound dehiscence	49	30	1.633	1.041-2.562	.033*
Hematoma	10	12	0.833	0.361-1.924	.669
Seroma	32	16	2	1.101-3.633	.023*
Readmission	27	13	2.077	1.075-4.014	.030*
Inpatient hospitalization	65	52	1.250	0.873-1.791	.224
Hematoma/seroma evacuation	38	19	2	1.157-3.457	.013*
Opioid prescription	1005	723	1.390	1.292-1.496	<.001*
Any surgical-site complications	164	71	2.310	1.761-3.031	<.001*

Ninety-day postoperative outcomes among patients undergoing abdominoplasty, comparing those receiving preoperative proton pump inhibitors (PPIs) with those in the control group receiving H2 blockers. Outcomes are reported as risk ratios (RRs) with 95% CIs.

^*^Statistically significant.

## DISCUSSION

**Figure 1.**
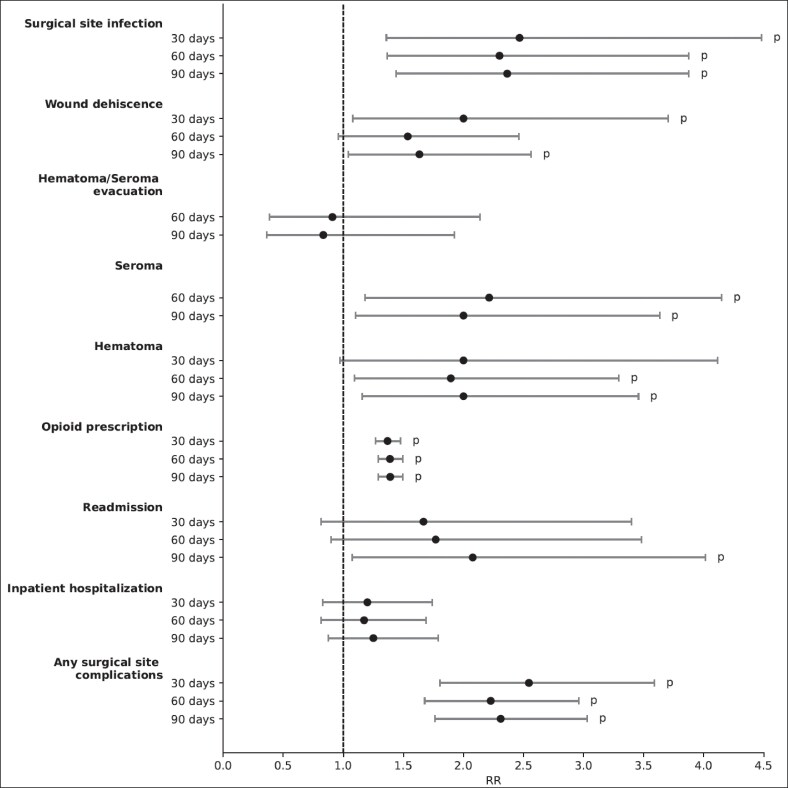
Risk ratio (RR) forest plot illustrating the risk of postoperative complications at 30, 60, and 90 days among patients undergoing abdominoplasty, comparing those who received preoperative proton pump inhibitors with those in the control cohort receiving H2 blockers. Statistically significant results were marked with “p.”

In this multicenter cohort study, preoperative use of PPIs among patients undergoing abdominoplasty was associated with a significantly increased risk of surgical-site complications, readmission, and opioid prescription (Figure 1). This association remained consistent across both measured time points, suggesting that PPI use may carry an impact on short-term postoperative complications following abdominoplasty.

Several proposed mechanisms may explain our findings, which are likely related to the systemic effect associated with prolonged PPI use. The first derives from the role of the microbiota in the development of surgical wound complications.^11,12^ It has been previously suggested that PPI use alter gastric pH and potentially disrupt the gut microbiome.^2,4,13^ By disrupting the gut microbiome and causing dysbiosis, PPIs may reduce microbial diversity and increase the abundance of opportunist pathogenic bacteria, consequently increasing the risk of surgical-site infections.^13-15^ Although the relationship with the gut microbiome may appear at first unrelated, a recent study utilizing the same worldwide database as ours (TriNetX) demonstrated that PPI use increases the risk of cutaneous infections, underscoring the impact of gut dysbiosis on skin-related conditions.^16^

Another potential mechanism relates to the effect of PPIs on gastric acid secretion, which may interfere with the absorption of certain nutrients.^17-19^ Because nutrients play an important role in collagen synthesis and immune function during the wound-healing process, impaired absorption may hinder proper and efficient healing.^20-22^ This, in turn, may be exacerbated by the effects of PPI use on inflammatory pathways and immune cells’ function. Several studies suggest that PPIs may weaken the immune response by impairing neutrophil function, affecting their bacterial-defense mechanisms, and inhibiting lysosomal enzyme activity.^23-25^ These, collectively, may impact bacterial-defense mechanisms and delay the wound-healing process, thereby increasing the risk of postoperative complications.

Our finding that patients in the PPI group exhibited high rates of opioid prescription during the early postoperative period suggests a potential link between PPI use and pain regulation or opioid tolerance. Although a review of current literature does not provide direct evidence for this association, an indirect mechanism may be involved. As discussed above, prolonged use of PPIs has been shown to induce gut dysbiosis, by increasing the migration of oral commensals downward the gastrointestinal tract.^13,14^ Interestingly, distinct reports have accumulated suggesting that dysbiosis may accelerate morphine tolerance as well, suggesting a potential link that may influence morphine efficacy.^26^ Alternatively, studies suggest that gut microbiota may be linked to chronic pain and opioid use, by altering immune and inflammatory components involved in pain processing.^27-29^ Yet, it is important to acknowledge that our findings do not directly measure opioid use, but rather reflect prescriptions of these medications, and therefore should be interpreted with caution. To our knowledge, the association between PPI-induced dysbiosis and opioid or pain modulation has not been directly examined. Future studies should investigate this association.

Our study has several limitations. First, its retrospective design and reliance on electronic medical record data from a large, multi-institutional database. Although this methodology, alongside the use of propensity score matching, may reduce confounding and balance patient characteristics, it introduces potential biases related to data accuracy and consistency. Second, we grouped all PPIs into a single category, despite differences in their pharmacologic properties, which may affect patients differently. Future studies should therefore evaluate the effect of PPIs with more detailed stratification. Additionally, we lack data on medication adherence, surgical techniques, follow-up, and diagnoses from institutions outside the TriNetX network. Furthermore, although several postoperative risks reached statistical significance, the absolute number of events was relatively small; therefore, further research is warranted to validate these findings. Patients using H2 blockers were selected as controls rather than patients without any acid-suppressive therapy. Because both groups share overlapping comorbidities, this approach enhanced comparability and helped minimize confounding; however, it is important to note that the comparison reflects the relative risk of PPI use among patients with these acid-related conditions rather than risk compared with the broader abdominoplasty population. Lastly, postoperative opioid prescriptions reflect prescribing patterns rather than consumption. Prescription behavior may be influenced by surgeon preference and institutional protocols, and therefore findings related to opioid prescriptions should be interpreted cautiously.

Our findings may have several clinical implications. Both surgeons and patients should be aware of the potential postoperative risks associated with preoperative PPI use. Yet we acknowledge that given that the absolute risks were relatively low, discontinuation of these medications perioperatively should not be routine and need to be guided individually through shared decision making. For those who continue PPI therapy, closer postoperative monitoring and wound care may be beneficial in mitigating potential complications. Our findings highlight the need for further research that accounts for different medication regimens and PPI types to better evaluate postoperative risk in this population.

## CONCLUSIONS

This large multicenter cohort study found that preoperative PPI use was associated with an increased risk of short-term postoperative complications. These findings highlight the need for close monitoring of abdominoplasty candidates with preoperative PPI exposure.

## Supplemental Material

This article contains supplemental material located online at https://doi.org/10.1093/asjof/ojag129.

## Supplementary Material

ojag129_Supplementary_Data
