# Supplementary material for: Association Between Proton Pump Inhibitor Use and Short-Term Postoperative Complications Following Abdominoplasty: A Multi-Institutional Cohort Study
Source: Aesthet Surg J Open Forum. 2026 Jun 23;8:ojag129. doi: 10.1093/asjof/ojag129 (PMC13344097; doi:10.1093/asjof/ojag129)
Supplement: ojag129_Supplementary_Data [file ojag129_supplementary_data.docx]

**Supplementary Table 1**: Current Procedural Terminology (CPT), Anatomical Therapeutical Chemical (ACT), and International Classification of Diseases, Revision 10 (ICD-10) codes that were used for patient selection.

| **Surgical Procedure** | **CPT Code** |
| --- | --- |
| Abdominoplasty | CPT:15847; SNOMED:177250006 |
| **PPIs cohort** |  |
| **Must have:**  omeprazole (RXNORM:7646)  pantoprazole (RXNORM:40790)  esomeprazole (RXNORM:283742)  lansoprazole (RXNORM:17128)  rabeprazole (RXNORM:114979) | |
| **H2 cohort** |  |
| **Cannot have:**  omeprazole (RXNORM:7646)  pantoprazole (RXNORM:40790)  esomeprazole (RXNORM:283742)  lansoprazole (RXNORM:17128)  rabeprazole (RXNORM:114979)  **Must have:**  famotidine (RXNORM:4278)  ranitidine (RXNORM:9143) | |
| **Outcomes & Complications** | **ICD-10 and CPT Codes** |
| Surgical site infection | ICD10CM: T81.4, T81.4XXA |
| Wound dehiscence | ICD10CM:T81.30, T81.31, T81.32; ICD9CM:998.3 |
| Hematoma | ICD10CM: M96.840, M96.841, L76.31, L76.32, M79.81 |
| Seroma | ICD10CM: L76.34, L76.33, M96.842, M96.843 |
| Readmission | CPT: 99221, 99222, 99223 |
| Inpatient hospitalization | CPT: 1013659, 10140, 1021538 |
| Hematoma/seroma evacuation | CPT:10160 |
| Opioid use | VA:CN101 |
| Any surgical site complications | ICD10CM: T80-T88 |

**Supplementary Table 2**: variables of the Propensity score matching

| **Code** | **Description** |
| --- | --- |
| I10-I1A | Hypertensive diseases |
| E10 | Type 1 diabetes mellitus |
| E11 | Type 2 diabetes mellitus |
| E66 | Overweight and obesity |
| F17 | Nicotine dependence |
| K21 | Gastro-esophageal reflux disease |
| K27 | Peptic ulcer, site unspecified |
| K92.0 | Hematemesis |
| N18 | Chronic kidney disease (CKD) |
| J44 | Other chronic obstructive pulmonary disease |
| 9083 | BMI |
